# Postinfusion Phlebitis: Incidence and Risk Factors

**DOI:** 10.1155/2015/691934

**Published:** 2015-05-14

**Authors:** Joan Webster, Matthew McGrail, Nicole Marsh, Marianne C. Wallis, Gillian Ray-Barruel, Claire M. Rickard

**Affiliations:** ^1^Centre for Clinical Nursing, Royal Brisbane and Women's Hospital, Herston, QLD 4029, Australia; ^2^National Health and Medical Research Council Centre of Research Excellence in Nursing Interventions for Hospitalised Patients, Griffith Health Institute, Nathan, QLD 4029, Australia; ^3^School of Rural Health, Monash University, Churchill Campus, Churchill, VIC 3842, Australia; ^4^Alliance for Vascular Access Teaching and Research (AVATAR), Griffith Health Institute, Nathan, QLD 4558, Australia; ^5^School of Nursing and Midwifery, University of the Sunshine Coast, Sippy Downs, QLD 4558, Australia

## Abstract

*Objective.* To document the incidence of postinfusion phlebitis and to investigate associated risk factors. *Design.* Analysis of existing data set from a large randomized controlled trial, the primary purpose of which was to compare routine peripheral intravascular catheter changes with changing catheters only on clinical indication. *Participants and Setting.* Patients admitted to a large, acute general hospital in Queensland, Australia, and who required a peripheral intravenous catheter. *Results.* 5,907 PIVCs from 3,283 patients were studied. Postinfusion phlebitis at 48 hours was diagnosed in 59 (1.8%) patients. Fifteen (25.4%) of these patients had phlebitis at removal and also at 48 hours after removal. When data were analyzed per catheter, the rate was lower, 62/5907 (1.1%). The only variable associated with postinfusion phlebitis was placement of the catheter in the emergency room (*P* = 0.03). *Conclusion.* Although not a common occurrence, postinfusion phlebitis may be problematic so it is important for health care staff to provide patients with information about what to look for after an intravascular device has been removed. This trial is registered with ACTRN12608000445370.

## 1. Introduction

Peripheral intravascular catheterization (PIVC) is a common feature of acute hospitalization, with the majority of patients requiring the intravenous administration of fluid or medication at some time during their hospital stay [1, 2]. One of the complications of PIVC is phlebitis, diagnosed by one or more signs or symptoms of pain, tenderness, swelling, induration, erythema, and a palpable, cord-like vein [3]. A number of factors have been associated with the development of phlebitis, such as (1) chemical factors—caused by irritant drugs or infusates; (2) mechanical factors—size, location and catheter material, and skill of the inserter; (3) infection factors—migration of organisms from the skin, along the catheter to the tip or from a contaminated hub; and (4) patient factors—infection at another location, age, and gender [4]. Irrespective of the cause, phlebitis may extend the patient's length of hospital stay, increase treatment costs, and, in rare cases, lead to bacteremia [5].

At least 71 different phlebitis scales exist [3]; consequently, the incidence of phlebitis varies widely, depending on the population studied and the definitions used for diagnosis. For example, rates as high as 91% have been reported in older studies [6] but results from recent large trials suggest that the percatheter incidence of phlebitis in tertiary hospitals is more likely to be around 4.6% [7], close to the recommended target of 5% set by the Infusion Nurses Society [8]. Most cases of phlebitis are minor and resolve without treatment when the catheter is removed. However, reported rates are generally based on phlebitis occurring during the course of intravenous therapy, whereas phlebitis is an inflammatory response and may occur well after the device is removed [9].

Although the Infusion Nurses Society Standards recommend that the vascular access site should be monitored for 48 hours following removal of the catheter to identify complications [8], little is known about the incidence of postinfusion phlebitis. Only two studies reported postinfusion phlebitis separately from phlebitis occurring during therapy, but results from the two studies were contradictory. In the first study, 445 patients from a North American acute general hospital were followed for 48 hours after the catheter was removed. Although plastic catheters were used in all but 1% of patients, the incidence of phlebitis in this cohort was 40% [9]. In the second study, also conducted by an acute health-care facility in North America, the postinfusion rate among 305 acute care patients was 1.0% [10]. The former study may indicate that phlebitis rates are hugely underreported if postcatheter removal follow-up is not conducted or that the high rate was an artefact of this 30-year-old study. Either way, there remains a dearth of information about the incidence of postinfusion phlebitis and the associated risk factors. This prevents reliable advice being given to nurses and patients about this complication. Consequently, data from our recent randomized controlled trial, which collected phlebitis data up to 48 hours after catheter removal, was reviewed.

## 2. Methods

### 2.1. Study Design and Participants

A post hoc analysis of a multicenter randomized controlled trial (Australian New Zealand Clinical Trials Registry, ACTRN12608000445370), which was designed to compare routine removal and replacement of intravenous catheters with replacement when clinically indicated was conducted. Details of the trial are published elsewhere [7] but, in brief, 3283 adult patients admitted to medical or surgical wards of three university-affiliated government hospitals in Queensland, Australia, and who required a peripheral intravenous catheter for at least four days were eligible. Exclusion criteria were blood stream infection, planned removal of the catheter within 24 hours, or intravenous catheter* in situ* for more than 72 hours. About 40% of catheters were inserted by an IV team with others inserted by the hospital's medical and nursing staff. Ethics approval was provided by Griffith University and each of the study hospitals. Patients provided written, informed consent [7].

### 2.2. Randomization and Interventions

Patients, stratified by hospital, were allocated (1 : 1 ratio) to the intervention or control group using a computer-generated, hand-held device, which concealed allocation until each patient's study entry. Masking was not possible after randomisation because of the nature of the intervention. Patients in the clinically indicated group had their catheters removed only on completion of therapy or if a complication occurred, such as phlebitis, infiltration, occlusion, or accidental removal. Those in the routine replacement group had their catheters removed and replaced every third day, unless replacement was indicated by catheter-related complication.

### 2.3. Outcomes and Follow-Up

The primary outcome for the original study was phlebitis during the course of intravenous therapy or within 48 hours after removal. Definitions used to diagnose phlebitis have been published in detail but included two or more of the following, occurring simultaneously: pain or tenderness rated >1 out of 10, erythema >1 cm from insertion site, swelling, purulent discharge, or palpable venous cord [7]. The insertion site was inspected daily by research nurses and at 48 hours after removal. If the patient had been discharged from hospital before 48 hours after cannula removal, follow-up was by telephone.

### 2.4. Analysis

The observed number of positive cases of postinfusion phlebitis is described as frequencies (%) and rates (1 in *n*). Bivariate associations between covariates and 48-hour postinfusion phlebitis were analyzed using the Chi-square statistic.

## 3. Results

During the study, 5,907 PIVCs from 3,283 patients were studied. The mean age of participants was 55.1 (SD 18.5), a total of 2,056 (62.6%) were male, 2,660 (81.0%) were admitted for surgery, and 2,485 (75.7%) reported having at least one comorbidity. Postinfusion phlebitis at 48 hours was diagnosed in 59 (1.8%) patients. Fifteen (25.4%) of these patients had phlebitis at removal and also at 48 hours after removal. In three of the total cohort, two cases of postinfusion phlebitis were recorded; so, when data were analyzed per catheter, the rate was lower, 62/5907 (1.1%). Pain and tenderness were the symptoms most frequently reported; rates of other signs and symptoms are reported in Table 1. The only variable associated with postinfusion phlebitis in the bivariate analysis was the location of catheter insertion: ward 46/4469 (1.03%), emergency department 12/569 (2.07%), operating room/radiology 4/474 (0.54%), and other locations 0/110 (0.0%); *P* = 0.03. A logistic regression analysis to identify factors associated with postinfusion phlebitis was planned but this was not sensible with such a low “event” rate and a large number of potential predictors.

## 4. Discussion

Results from the large RCT, with careful follow-up for at least 48 hours, closely correspond with findings from a similar study [10] and suggest that postinfusion phlebitis is not a common problem. Nevertheless, phlebitis may be a very painful condition and can take as long as seven days to resolve [9]. Additionally, in rare cases, phlebitis may be associated with a blood stream infection, a much more serious and potentially life-threatening condition [11]. Importantly, 75% of cases of postinfusion phlebitis were diagnosed in people who did not have phlebitis when the catheter was removed. Consequently, it is important for health care staff to provide patients with information about what to look for after an intravascular device has been removed. Advice about reporting any persistent problem to a nurse or medical practitioner or, following hospital discharge, to their general practitioner is also essential.

The only significant predictor of postinfusion phlebitis in this analysis was the location in which the catheter was inserted, reinforcing the recommendation that catheters placed in emergency situations should be removed and resited as soon as appropriate [12]. However, the definition of phlebitis used in the study was stringent, which may have underrepresented the incidence of catheter-related outcomes. For example, some participants had a pain score of 3 or 5, thus causing significant annoyance, discomfort, and sometimes catheter removal but failed to meet our criteria for phlebitis. It is also quite possible, if the incidence of postinfusion phlebitis had been greater, that other factors commonly associated with phlebitis at catheter removal, such as younger age, larger PIVC, female gender, coexisting infection, and type of infusate [4], may have also been predictive for postinfusion phlebitis in this study.

## 5. Conclusion

Although postinfusion phlebitis is an uncommon outcome following peripheral vascular catheterization, the access site should be observed for at least 48 hours after catheter removal to ensure appropriate management, if postinfusion phlebitis is identified.

## Figures and Tables

**Table 1:** Frequency of reported signs and symptoms of phlebitis at ≥48 hours after catheter removal (*N* = 5907 catheters).

Criteria	Number (%)	Occurrence
Pain/tenderness > 1 cm	55 (0.93)	1 in 107
Swelling	40 (0.68)	1 in 148
Erythema > 1 cm	27 (0.46)	1 in 219
Palpable cord	18 (0.30)	1 in 328
Purulent	2 (0.03)	1 in 2954
